# Aggression Traits in Youth Psychopathy: The Key Role of Serotonin

**DOI:** 10.3389/fpsyt.2014.00025

**Published:** 2014-03-17

**Authors:** Carmelo M. Vicario

**Affiliations:** ^1^School of Psychology, Bangor University, Bangor, UK

**Keywords:** psychopathic traits, childhood, development, serotonin, behavior

In a recent article, Blair (1) addresses the neurobiological mechanisms underlying psychopathic traits in childhood. The analysis conducted by the author provides a complete view about the complexity of neuro-functional factors underlying childhood psychopathy. In particular, the author focuses on the role played by structural, endocrinological, genetic, and environmental factors.

Surprisingly, this article offers limited attention to the growing body of evidence about the role of serotonin in aggression (2) and emotional empathy (3), two important aspects of psychopathy, although the author mentioned the effect of the serotonin transporter polymorphism in developing aggression (4) as well as the impact played by serotonin depletion in the recognition of fearful expressions [i.e., Ref. (5)].

The literature provides strong support to the suggestion that the pediatric origin of psychopathy might be grounded on serotoninergic abnormalities. Kruesi et al. (6) examined the cerebrospinal fluid levels of 5-hydroxyindoleacetic acid, a metabolite of serotonin, in relation to aggression, impulsivity, and social functioning in 29 children and adolescents with disruptive behavior disorders. The cerebrospinal fluid 5-hydroxyindoleacetic acid level was low compared with that of age-, sex-, and race-matched patients with obsessive–compulsive disorder.

Further insights are also provided by the study of borderline personality disorder (BPD), which onset might occur during childhood and adolescence (7). This disorder is characterized by impulsive aggressive behaviors, such as physical aggression directed toward others, self-mutilation, and domestic violence (8). Soloff et al. (9) have reported that patients with BPD have diminished response to serotonergic stimulation in areas of prefrontal cortex.

A relationship between serotoninergic abnormalities and aggression has been reported also in patients with anorexia nervosa (AN), an eating disorder typical of the pre-puberal and adolescence period (10). This disorder is characterized by marked disgust sensitivity for food (11, 12), but also by impulsivity and aggressive behavior. For example, Fava et al. (13) reported that the 28% of AN suffers have experienced uncontrollable anger attack compared with four (10%) of the control subjects. These symptoms can be explained in relation to a reduced serotonin activity in underweight patients with AN (14). The increased aggressive behavior in this clinical population could be indirectly caused by a lack of tryptophan (TRP), which is involved in the synthesis of serotonin (15). In fact, subjects on hypocaloric diets have reduced serotonin activity and show reduced TRP availability (16). Moreover, acute TRP depletion has been associated to aggression in young people with ADHD (17), while it has been reported that TRP integration contributes to behavioral regulation in boys with history of behavior regulation difficulties.

An indirect evidence in support of the current issue is also provided by role played by genetic variation of the serotonin transporter-linked polymorphic region (5-HTTLPR) in the connectivity of two key regions of psychopathy in youths (1) such as the amygdale (18) and the anterior cingulate cortex (19).

Finally, serotoninergic abnormalities may be called into question to explain the effects of negative experiences, such as childhood traumatization, in developing psychopathic traits. This is suggested by research on sexual abuse. Sexually abused women homozygous for the low-activity monoamine oxidase A allele, a gene involved in the synthesis of serotonin (20), had high rates of antisocial symptoms as compared to sexually abused woman who are homozygous for the high activity allele (21). According to this evidence, one could argue that serotonin plays a role not only with respect to a specific episode of violence and/or aggression (i.e., related to a particular context or state) but it represents an element of predisposition, which might actively contribute to the inclusion and consolidation of violence and aggression as a trait of personality, if exposed to particular (i.e., negative) experience. In this sense, serotonin can be considered relevant at trait level as suggested by Sadeh et al. (22).

Overall, the evidence discussed above provides support to the key role of serotonin in explaining the neurobiological origin of psychopathic symptoms in youths such as aggression. This is suggested not only by the direct effect of this monoamine on social behavior but also by its role, at genetic level, in determining the vulnerability to environmental experiences associated to antisocial and aggressive conducts. However, one must keep in mind that antisocial behavior is not synonymous of psychopathy (23, 24). Indeed, as noted by Hare (25), about the 90% of psychopathic aggressors meet criteria for antisocial personality disorders (APD), but only the 25% of individuals diagnosed with APD are psychopaths. Moreover, although the main goal of this article is to highlight the role of serotonin in aggressive behavior, it should be clarified that this tract can be influenced by other neurochemical factors. In this regard, the nitric oxide (NO) seems to play an important role (26). For example, it has been shown that the absence of the gene for the synthesis of the nitric oxide increases aggression in mice (27). This evidence extends the spectrum of the neurochemical factors involved in aggression and, at the same time, provides an explanation to the negative studies that has not reported a direct relationship between low levels of serotonin and aggression [i.e., Ref. (28)].

Finally, in the context of serotonin, it is important to provide a distinction between “impulsive aggression” and “premeditate aggression.” In fact, as reported by Linnoila et al. (29), serotonin was significantly lower in subjects affected by “impulsive violence” while the levels of this monoamine did not change significantly in the “premeditated violence” sample. This suggests that serotonin seems mainly implied in the impulsivity associated to aggression rather than aggression itself.

In summary, we have seen that serotonin appears to be of paramount importance in explaining aggression as trait of personality rather than as a reaction elicited by the context.
